# Penile Strangulation by a Plastic Band With an Unknown Time of Onset: A Report of a Rare Case

**DOI:** 10.7759/cureus.71596

**Published:** 2024-10-16

**Authors:** Ryo Sato, Asuka Uchiyama, Shungo Kakinuma, Rikiya Matsumoto

**Affiliations:** 1 Department of Urology, Chutoen General Medical Center, Kakegawa, JPN; 2 Department of Urology, Hamamatsu University School of Medicine, Hamamatsu, JPN

**Keywords:** intellectual disability (id), penile injury, penile strangulation, penile trauma, urological emergency

## Abstract

We report a case of penile strangulation by a plastic band with an unknown time of onset. A 61-year-old man was brought to the emergency department with complaints of painful edematous swelling of the penis caused by a plastic band. The patient had an intellectual disability and, thus, difficulties were associated with obtaining a detailed medical history, including the time of onset. Penile strangulation was released by surgical scissors in the emergency room. The patient made a satisfactory recovery seven days after admission, and no major complications were observed.

## Introduction

Penile strangulation is a relatively rare urological emergency situation that requires proper immediate management because longer strangulation times are associated with more severe complications, such as penile gangrene, urethral fistula, and stricture [1]. Previous studies suggested that penile strangulation occurs due to attempts to achieve sexual pleasure, erection reinforcement, or secondary to psychiatric disorders [1,2]. We herein report a case of a 61-year-old male with intellectual disability who presented to the emergency ward with penile strangulation by a plastic band at an unknown time of onset.

## Case presentation

**Figure 1:**
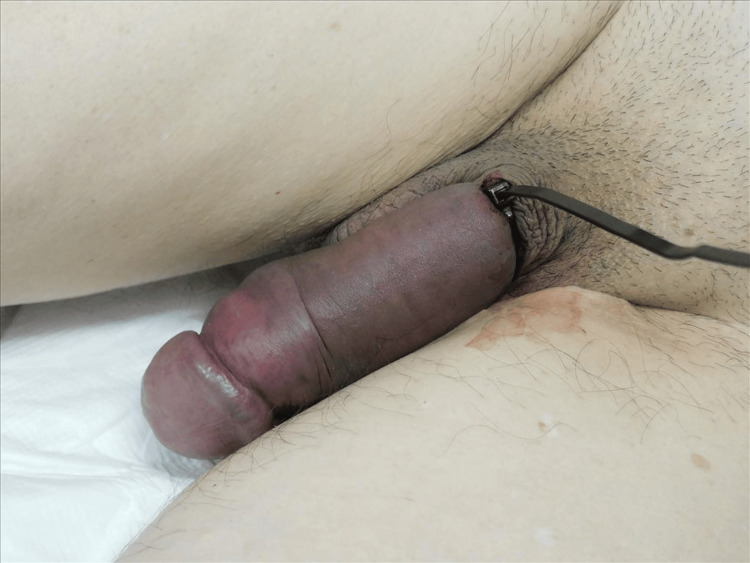
Penile ecchymosis and edema (dorsal view)

**Figure 2:**
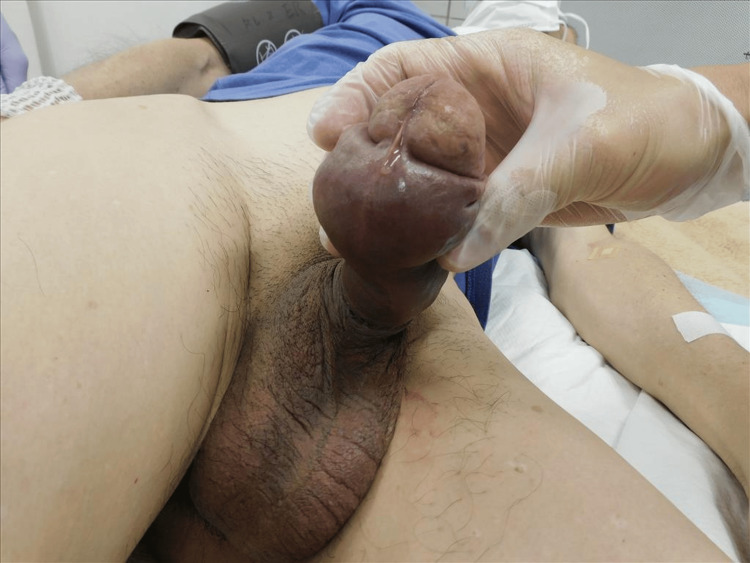
Penile ecchymosis and edema (ventral view)

A 61-year-old man was brought to the emergency department with complaints of painful edematous swelling of the penis caused by a plastic band. The patient developed these symptoms after the application of a plastic band. The patient had a history of intellectual disability with an estimated intelligence quotient of 40 and was living in a social welfare facility. As a result, it was difficult to obtain a detailed medical history, such as the motivation for penile strangulation and the time of onset. According to the facility staff, there were no previous signs of self-harm or attempts to constrict the penis. A physical examination revealed penile lacerations at the band site along with edema and ecchymosis (Figures 1-2). Furthermore, the patient developed urinary retention secondary to severe penile edema. The emergency team tried to remove the plastic band with surgical scissors and was successful. A 14-fr Foley urethral catheter was inserted to empty the bladder, and clear urine was observed. The patient was admitted to hospital and given antibiotics. The catheter was taken out on day 4 of admission; after seven days of hospitalization, the patient was discharged with reduced penile edema, clean wounds, and normal urinary status. All treatment was performed after obtaining informed consent from the guardian.

## Discussion

Penile strangulation was initially described by Gauthier in the 18th century [3]. Since then, there have been several reports of strangulation by metallic or non-metallic objects. Zeid et al. reported a case of penile entrapment by a metal ring [4]. In addition, Rohith et al. reported a case of penile strangulation by a plastic bottleneck [5]. Penile strangulation caused by metallic objects may be difficult to remove. In the report by Zeid et al., it took more than five hours to remove the metal ring [4]. Moreover, Nguyen et al. reported a case of penile strangulation caused by a steel nut, in which standard bolt cutters were not effective but a dental handpiece was effective for removal [6]. In the present case, the plastic band, a non-metallic object, was easily removed.

In 1991, Bhat et al. established a classification for penile strangulation consisting of five grades based on the degree of penile injury [7]. Silberstein et al. simplified and modified the proposed classification into two broad categories (Table 1) [1]. In the Silberstein classification, low-grade penile injuries correspond to Bhat grade 1-3 injuries and do not require further intervention in most cases after removal of the foreign body. On the other hand, high-grade penile injuries correspond to Bhat grade 4 and 5 injuries and typically require surgical intervention. The present case was considered a Grade 2 Bhat grade or low-grade penile injury in the Silberstein grades based on the findings on the penis and, thus, was conservatively managed.

**Table 1:** Grading systems of penile strangulation

Grade	Penile injury grading system by Bhat et al. [7]	Revised grading system by Silberstein et al. [1]
1	Edema of the distal penis. No evidence of skin ulceration or urethral injury.	Low-grade penile injury
2	Injury to skin and constriction of the corpus spongiosum, but no evidence of urethral injury. Distal penile edema with decreased penile sensation.	
3	Injury to skin and the urethra, but no urethral fistula. Loss of distal penile sensation.	
4	Complete division of the corpus spongiosum leading to urethral fistula and constriction of the corpus cavernosum with the loss of distal penile sensation.	High-grade penile injury
5	Gangrene, necrosis, or complete amputation of the distal penis.	

Silberstein et al. reported a higher incidence of significant complications, such as penile gangrene, urethral fistula, and stricture, in patients presenting after 72 hours (29.1%) than in those presenting within 72 hours (0.0%) [1]. Koifman et al. reported a case in which conservative treatment was administered to a patient who presented to the hospital 72 hours after penile strangulation by a plastic ring but ultimately required partial penectomy [2]. In the present case, the exact onset time of penile strangulation was unknown due to the patient’s intellectual disability. However, based on the findings on the penis, surgical intervention was deemed unnecessary, and the patient had a good outcome.

In patients with intellectual disabilities, such as in the present case, reviewing the living environment and informing those involved will be helpful for the prevention of further episodes.

## Conclusions

Penile strangulation is a rare urological emergency. Even if the time of onset is unknown, it is important to carefully select the treatment strategy for penile strangulation based on penile findings. In patients with intellectual disabilities, reviewing the living environment and informing those involved is crucial to avoiding future incidents.
